# Incidence, Recurrence, and Risk Factors for Peri-ictal Central Apnea and Sudden Unexpected Death in Epilepsy

**DOI:** 10.3389/fneur.2019.00166

**Published:** 2019-03-01

**Authors:** Laura Vilella, Nuria Lacuey, Johnson P. Hampson, M. R. Sandhya Rani, Kenneth Loparo, Rup K. Sainju, Daniel Friedman, Maromi Nei, Kingman Strohl, Luke Allen, Catherine Scott, Brian K. Gehlbach, Bilal Zonjy, Norma J. Hupp, Anita Zaremba, Nassim Shafiabadi, Xiuhe Zhao, Victoria Reick-Mitrisin, Stephan Schuele, Jennifer Ogren, Ronald M. Harper, Beate Diehl, Lisa M. Bateman, Orrin Devinsky, George B. Richerson, Adriana Tanner, Curtis Tatsuoka, Samden D. Lhatoo

**Affiliations:** ^1^Department of Neurology, University of Texas Health Science Center at Houston, Houston, TX, United States; ^2^Epilepsy Center, University Hospitals Cleveland Medical Center, Cleveland, OH, United States; ^3^Department of Neurology, Case Western Reserve University, Cleveland, OH, United States; ^4^Department of Electrical Engineering and Computer Science, Case Western Reserve University, Cleveland, OH, United States; ^5^Department of Neurology, University of Iowa School of Medicine, Iowa City, IA, United States; ^6^NYU Langone School of Medicine, New York, NY, United States; ^7^Sidney Kimmel Medical College, Thomas Jefferson University, Philadelphia, PA, United States; ^8^Division of Pulmonary, Critical Care and Sleep Medicine, University Hospitals Medical Center, Cleveland, OH, United States; ^9^Institute of Neurology, University College London, London, United Kingdom; ^10^Feinberg School of Medicine, Northwestern University, Chicago, IL, United States; ^11^Department of Neurobiology and the Brain Research Institute, University of California, Los Angeles, Los Angeles, CA, United States; ^12^Department of Neurology, Columbia University, New York, NY, United States; ^13^Mercy Health St. Mary's Campus, Grand Rapids, MI, United States

**Keywords:** apnea, breathing, epilepsy, ictal central apnea (ICA), seizures, sudden unexpected death in epilepsy (SUDEP), post-convulsive central apnea (PCCA)

## Abstract

**Introduction:** Peri-ictal breathing dysfunction was proposed as a potential mechanism for SUDEP. We examined the incidence and risk factors for both ictal (ICA) and post-convulsive central apnea (PCCA) and their relationship with potential seizure severity biomarkers (i. e., post-ictal generalized EEG suppression (PGES) and recurrence.

**Methods:** Prospective, multi-center seizure monitoring study of autonomic, and breathing biomarkers of SUDEP in adults with intractable epilepsy and monitored seizures. Video EEG, thoraco-abdominal excursions, capillary oxygen saturation, and electrocardiography were analyzed. A subgroup analysis determined the incidences of recurrent ICA and PCCA in patients with ≥2 recorded seizures. We excluded status epilepticus and obscured/unavailable video. Central apnea (absence of thoracic-abdominal breathing movements) was defined as ≥1 missed breath, and ≥5 s. ICA referred to apnea preceding or occurring along with non-convulsive seizures (NCS) or apnea before generalized convulsive seizures (GCS).

**Results:** We analyzed 558 seizures in 218 patients (130 female); 321 seizures were NCS and 237 were GCS. ICA occurred in 180/487 (36.9%) seizures in 83/192 (43.2%) patients, all with focal epilepsy. Sleep state was related to presence of ICA [RR 1.33, CI 95% (1.08–1.64), *p* = 0.008] whereas extratemporal epilepsy was related to lower incidence of ICA [RR 0.58, CI 95% (0.37–0.90), *p* = 0.015]. ICA recurred in 45/60 (75%) patients. PCCA occurred in 41/228 (18%) of GCS in 30/134 (22.4%) patients, regardless of epilepsy type. Female sex [RR 11.30, CI 95% (4.50–28.34), *p* < 0.001] and ICA duration [RR 1.14 CI 95% (1.05–1.25), *p* = 0.001] were related to PCCA presence, whereas absence of PGES was related to absence of PCCA [0.27, CI 95% (0.16–0.47), *p* < 0.001]. PCCA duration was longer in males [HR 1.84, CI 95% (1.06–3.19), *p* = 0.003]. In 9/17 (52.9%) patients, PCCA was recurrent.

**Conclusion:** ICA incidence is almost twice the incidence of PCCA and is only seen in focal epilepsies, as opposed to PCCA, suggesting different pathophysiologies. ICA is likely to be a recurrent semiological phenomenon of cortical seizure discharge, whereas PCCA may be a reflection of brainstem dysfunction after GCS. Prolonged ICA or PCCA may, respectively, contribute to SUDEP, as evidenced by two cases we report. Further prospective cohort studies are needed to validate these hypotheses.

## Introduction

Sudden Unexpected Death in Epilepsy (SUDEP) is the leading cause of premature mortality in patients with intractable epilepsy (1). The main SUDEP phenotype comprises frequent generalized convulsive seizures in patients with early onset, longstanding epilepsy (2, 3). Both cardiac and respiratory mechanisms likely contribute to SUDEP pathophysiology (3, 4), although video electroencephalogram monitored (VEEG) deaths suggest that terminal cardiac arrest is almost always preceded by central apnea (5). Central, obstructive and mixed apneas have all been proposed as SUDEP mechanisms, and may occur during or after seizures (6–9). Whereas, ictal central apnea (ICA) is common, prolonged ICA with profound oxygen desaturation may pose SUDEP risk, as may post-convulsive central apnea (PCCA) (10–14). The latter, when combined with bradycardia/asystole, comprised the majority of observed deaths in the MORTEMUS series, and two near-SUDEP instances in one observational SUDEP risk study (14). Thus, it is evident that breathing dysfunction plays a major role in SUDEP, although the exact characteristics of respiratory compromise that contribute to death are unknown (15). Since central apnea (prolonged ictal or post-convulsive) seems a viable, agonal mechanism, we set out to determine incidence, recurrence, and characteristics of peri-ictal central apnea. We assessed its influence on potential seizure severity biomarkers, such as hypoxemia extent and post-ictal generalized electroencephalographic (EEG) suppression (PGES) in a prospective study of SUDEP risk biomarkers. Further, we describe two additional cases of near-SUDEP due to prolonged, exaggerated peri-ictal central apnea.

## Material And Methods

### Patient Selection

All patients were prospectively recruited participants in the NINDS Center for SUDEP Research's Autonomic and Imaging Biomarkers of SUDEP multi-center project (U01-NS090407), and its preliminary phase, the Prevention and Risk Identification of SUDEP Mortality (PRISM) Project (P20NS076965). This study was carried out in accordance with the recommendations of University Hospitals Case Medical Center Institutions Review Boards (UHIRB) and University of Iowa, School of Medicine, Iowa City (IA) Institutions Review Boards. The protocol was approved by UHIRB and University of Iowa, School of Medicine, Iowa City (IA) Institutions Review Boards. All subjects gave written informed consent in accordance with the Declaration of Helsinki. Informed written consent was obtained for publication of two clinical cases. Patients with intractable epilepsy (failure of adequate trials of two or more antiepileptic medications) (16) aged ≥18 years underwent video-electroencephalography (VEEG) evaluation in the adult epilepsy monitoring units (EMU) of participating centers from September 2011 until April 2018. We included patients with recorded electroencephalographic seizures with or without clinical correlate, who had complete polygraphic physiological recordings sufficient for analysis. Exclusion criteria were status epilepticus (SE), obscured or unavailable video, and insufficient multimodal physiological recording quality.

Demographic and clinical data were collected. Semiology was classified into two types (17). (1) Generalized convulsive seizures (GCS) which included generalized tonic-clonic seizures, focal to bilateral tonic-clonic seizures, and focal onset motor bilateral clonic seizures. (2) Non-convulsive seizures (NCS), which included seizures with focal onset without evolution to bilateral tonic-clonic seizures, myoclonic seizures, absence seizures, and electroencephalographic seizures without clinical correlate. We determined state (awake or asleep) at seizure onset. We defined the putative epileptogenic zone based on clinical history, seizure semiology, neuroimaging, and VEEG.

### Data Collection

All patients underwent prolonged surface VEEG monitoring using the 10–20 International Electrode System. EEG and electrocardiogram (EKG) were acquired using Nihon Kohden (Tokyo, Japan), Micromed (Modigliani Veneto, Italy), and Xltek (Natus) acquisition platforms. Peripheral capillary oxygen saturation (SpO_2_) was monitored using pulse oximetry (Nellcor OxiMax N-600x [Convidien], Masimo Radical-7 [Irvine], and SenTec Digital Monitoring System [Therwil BL]) and plethysmography (Ambu [Ballerup, Denmark] Sleepmate and Perfect Fit 2 [Dymedix]). Chest wall and abdominal excursions were recorded using inductance plethysmography (Ambu, Ballerup, Denmark and Sleepmate or Perfect Fit 2, Dymedix, St Paul, MN, USA).

Breathing analysis for apnea utilized careful composite analysis of inductance plethysmography, EEG breathing artifact and visually inspected thoraco-abdominal excursions 2 min before seizure onset (clinical or electrographic, whichever that occurred first). Central apnea (cessation of thoraco-abdominal breathing movements) was defined as ≥1 missed breath without any other explanation (i.e., speech, movement, or intervention), with a minimal duration of 5 s. ICA referred to apnea in NCS, or apnea occurring in the pre-convulsive phase of GCS. PCCA referred to apnea after GCS; we preferred this term to post-ictal central apnea since it could occur after convulsions but before EEG seizure end. Incidences of ICA and PCCA were determined, as well as their durations. Apnea could not be, and was not assessed during tonic or clonic movements, because of invariable artifact presence in breathing channels. A subgroup analysis identified recurrences of ICA and PCCA (in ≥2 seizures).

Hypoxemia was defined as SpO_2_ < 95% and where baseline SpO_2_ was already <95% a >1% drop was considered significant. Oxygen desaturations were classified as mild (SpO2 90–94%), moderate (75–89%), and severe (<75%). For SpO_2_ evaluation, several time points were considered. Firstly, SpO_2_ was determined at baseline, 2 min pre-ictally as mean SpO_2_ in a 15 s, artifact free epoch. For GCS and NCS, the overall desaturation nadir referred to the lowest SpO_2_ value registered during and up to 3 min after the seizure. To evaluate respiration in the pre-convulsive phase of GCS, an additional desaturation nadir was considered during this phase in patients with ICA.

Presence and duration of PGES (18) after GCS was determined by a validated automated EEG suppression detection tool (19), and supplemented with visual analysis by two epilepsy neurophysiologists when the tool gave no solution. Presence and duration of post-ictal EEG burst suppression were also determined. Combined PGES and burst suppression comprised EEG “recovery” duration.

### Statistical Analysis

Statistical analyses were conducted using SPSS (version 24; IBM Corp, Armol, NY, USA) and SAS for Windows 9.4 (SAS Institute Inc., NC, USA). Summary statistics were reported as mean ± standard deviation (SD; median, range). Relative risk (RR) for the primary outcome of ICA and PCCA at a seizure level was assessed by Generalized Estimating Equation (GEE) with same subject exchangeable correlation. All variables with a *p* < 0.20 in a univariate analysis were included in a multivariate Poisson GEE regression (20). Variables related to ICA and PCCA durations were determined using Cox Regression with robust sandwich covariance estimation (21). Lastly, recurrence of ICA and PCCA for each patient with at least two seizures in this data were categorized as binary outcomes and patient-level covariates were included in respective logistic regressions. Corresponding 95% CIs of risk and hazard ratios were generated from these models. The significance level for hypothesis testing was set at *p* < 0.05.

## Results

### Demographic and Clinical Characteristics

Among 218 patients (130 female), 558 seizures met inclusion criteria. Four hundred and twenty-six seizures were previously reported in two different studies (13, 14).

Mean age at study was 40.2 years ± 14.7 (39; 18–77), mean epilepsy duration was 16.6 years ± 13.8 years (1 month−58 years) and mean age at epilepsy onset was 23.5 years ± 17.2 (20; 1 month-69 years). There were 321 NCS (in 128 patients) and 237 GCS (in 137 patients). State was sleep in 239 seizures and wakefulness in 318 seizures. One seizure arose from post-ictal coma in a patient with a seizure cluster.

There were 182 patients (493 seizures) with focal epilepsy and 33 patients (60 seizures) with generalized epilepsy. One patient had both focal and generalized epilepsy (2 seizures). Epilepsy type was unknown in 2 patients (3 seizures) (Table 1).

**Table 1:** Patient characteristics.

	**Number of seizures** **(*n* = 558)**	**Number of patients** **(*n* = 218)**
**SEX**		
Male	250	88
Female	308	130
**EPILEPSY TYPE**		
Generalized	60 (10.8%)	33 (15.1%)
Focal	493 (88.3%)	182 (83.5%)
Temporal	292 (52.3%)	115 (52.8%)
Frontal	90 (16.1)	33 (15.1%)
Multifocal	45 (8.1%)	15 (6.9%)
Lateralized	36 (6.5%)	11 (5%)
Occipital	11 (2%)	4 (1.8%)
Parietal	12 (2.2%)	2 (0.9%)
Insular	7 (1.3%)	2 (0.9%)
Focal and generalized	2 (0.4%)	1 (0.55)
Unknown	3 (0.5%)	2 (0.9%)
**LATERALIZATION**		
Right	169	70
Left	212	74
Bilateral	92	33
Generalized	60	33
Unknown	23	7
Focal and generalized	2	1

PGES could be determined in all but one GCS, where electrode artifact prevented accurate interpretation. PGES occurred in 165/236 (69.9%) GCS in 106/136 (77.9%) patients. Mean PGES duration was 38.9 s ± 21.2 (37; 1–169) and mean EEG recovery duration was 85 s ± 107.9 (54; 1–1,091).

### Ictal Central Apnea (ICA) Incidence, Duration, and Recurrence

ICA could not be confidently ascertained in 71 seizures, all GCS, due to plethysmographic signal acquisition artifact. ICA occurred in 180/487 (36.9%) seizures in 83/192 (43.2%) patients: 65/166 (39.2%) in GCS and 115/321 (35.8%) in NCS (*p* = 0.960).

**Figure 1:**
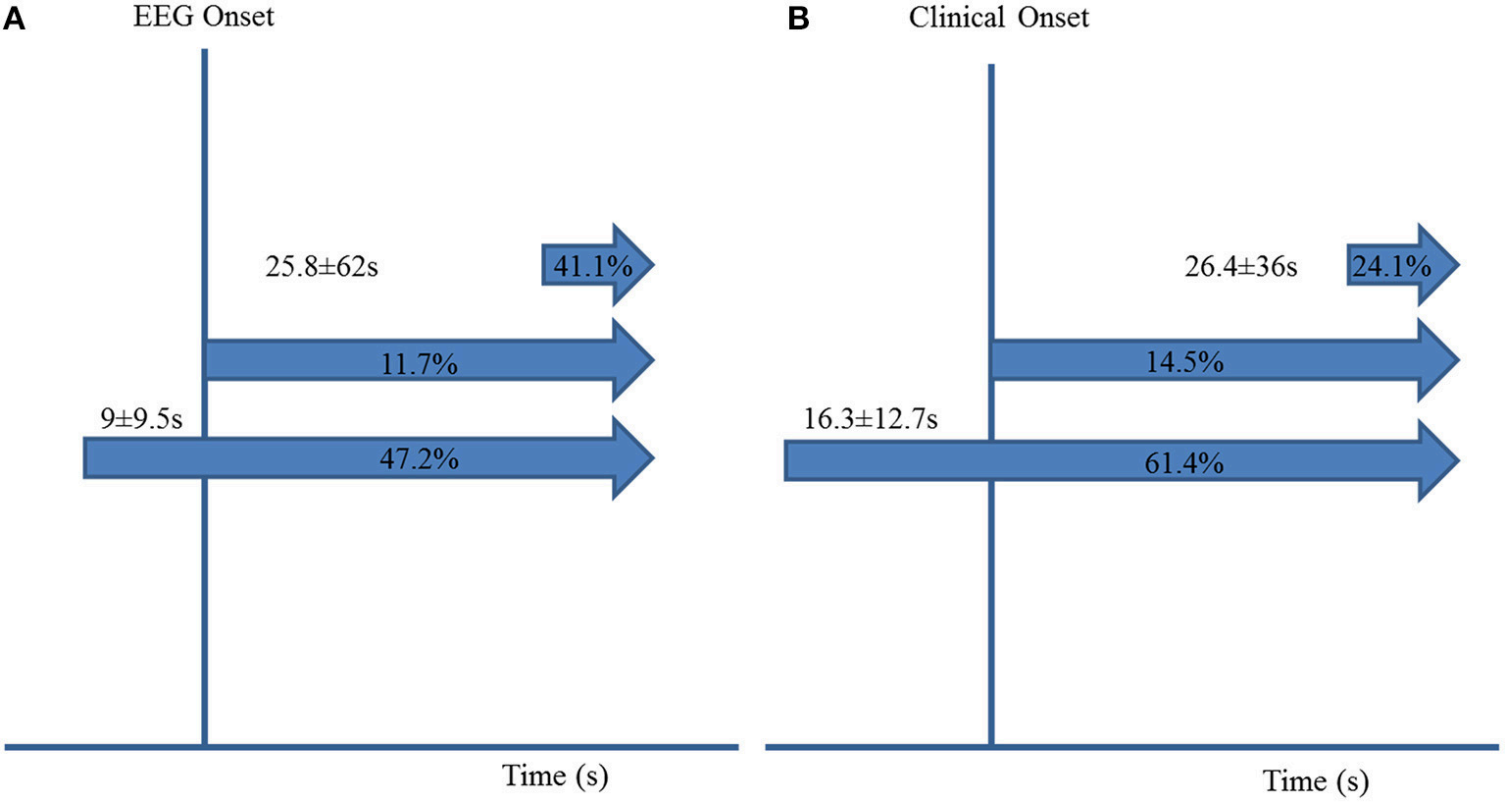
Ictal central apnea (ICA) timing with respect to EEG onset **(A)** and clinical onset **(B)**.

ICA preceded EEG seizure onset in 85/180 (47.2%) seizures by 9 s ± 9.5 (7; 1–58). ICA occurred after EEG seizure onset in 74/180 (41.1%) seizures, with a delay of 25.8 s ± 62 (8; 1–436). ICA coincided with EEG seizure onset in the remaining 21/180 (11.7%) seizures (Figure 1A).

**Figure 2:**
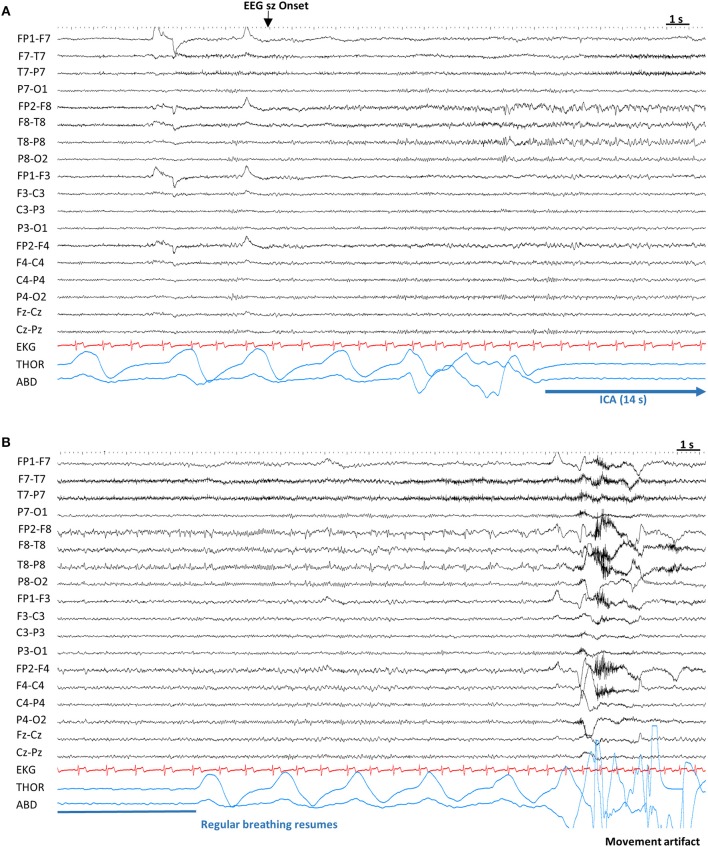
Example of ictal central apnea (ICA). Sensitivity 7 μV, High Frequency Filter: 70 Hz, Time constant: 0.1 s. **(A)** Twelve seconds after the electrographic onset, ICA is noted without any other clinical signs. **(B)** Continuation of ICA, with a total duration of 14 s, followed by breathing resumption. ABD, abdominal; EEG sz onset, electrographic seizure onset; EKG, electrocardiogram; ICA, Ictal central apnea; s, seconds; THOR, thoracic.

ICA was the sole manifestation in 14/180 (7.8%) seizures. It coincided with clinical onset in 24/166 (14.5%) seizures, started before clinical onset in 102/166 (61.4%), preceding it by 16.3 ± 12.7 s (12; 1–66 s), and started after clinical onset in 40/180 (24.1%) seizures, with a difference of 26.4 s ± 36 (14.5; 1–195) (Figures 1B, 2A,B).

Information regarding nadir SpO_2_ in NCS and the pre-convulsive phase of GCS was available in 141/180 (78.3%) seizures with ICA, with a mean value of 87.7% ± 9.4 (91; 46–99).

All 180 seizures with ICA were seen in focal epilepsies, and none in patients with generalized epilepsy. In patients with focal epilepsies ICA was more frequent in temporal lobe epilepsies than extratemporal (*p* = 0.002) but there was no association with laterality (*p* = 0.215). ICA incidence did not show any differences regarding sex (*p* = 0.171) or epilepsy duration (*p* = 0.077) but it was related to older age at study (*p* = 0.004) and older age at epilepsy onset (*p* < 0.001). ICA was more frequent in seizures arising from sleep than during wakefulness (*p* = 0.013). ICA was not related to PGES or PCCA and did not affect PGES, EEG recovery, hypoxemia or PCCA durations or SpO2 nadir in GCS (*p* > 0.050) (Table 2).

**Table 2:** ICA incidence and seizure characteristics.

			**Univariate analysis**	**Multivariate analysis**	
	**ICA–**	**ICA+**	***p***	**RR *(95% CI)***	***p***
Sex			0.171	0.87 (0.64–1.18)	0.383
Male	129	91			
Female	178	89			
Age at study (y.o)	38.2 ± 14.9	44.5 ± 15.3	0.004	–	–
Age at epilepsy onset (y)	18.7 ± 16.4	28 ± 17.1	<0.001	1.01 (1.00–1.02)	0.222
Epilepsy duration (y)	19.5 ± 14.1	16.6 ± 14.1	0.077	0.98 (0.97–1.00)	0.608
Epilepsy type			–	–	–
Generalized	51	0			
Focal	251	180			
Epileptogenic zone			0.002	0.58 (0.37–0.90)	0.015
Extratemporal	133	40			
Temporal	118	140			
Lateralization			0.215	–	–
Left	90	94			
Right	99	52			
State			0.013	1.33 (1.08–1.64)	0.008
Awake	191	92			
Asleep	116	87			
Semiology					
GCS	101	65	0.960	–	–
NCS	206	115			
PGES^a^			0.308	–	–
No	90	12			
Yes	70	53			
PGES duration^a^ (s)	36.3 ± 15.8	43.3 ± 29.7	0.618	–	–
EEG recovery duration (s)	70.9 ± 61.8	106.2 ± 162.1	0.512	–	–
Recovery time to mild hypoxemia^a^ (s)	41.8 ± 31.9	48.5 ± 46	0.903	–	–
Total hypoxemia duration^a^ (s)	147.5 ± 70.4	149.9 ± 56.2	0.953	–	–
SpO_2_ nadir^a^ (%)	59.5 ± 14.4	58.3 ± 13.2	0.576	–	–
PCCA^a^			0.785	–	–
No	79	49			
Yes	18	14			
PCCA duration^a^ (s)	8 ± 3.3	10.4 ± 6.7	0.509	–	–

*GCS, generalized convulsive seizures; NCS, non-convulsive seizures; PCCA, post-convulsive central apnea; PGES, post-ictal generalized electroencephalographic suppression; SpO_2_, peripheral capillary oxygen saturation; y, years y.o, years old*.

a *Analyzed only in GCS*.

After multivariate analysis, sleep state was related to presence of ICA [RR 1.326, CI95% (1.075–1.637), *p* = 0.008] whereas extratemporal epilepsy was related to lower incidence of ICA [RR 0.579, CI 95% (0.373–0.900), *p* = 0.015].

Mean ICA duration was 20.9 s ± 17.5 (14; 5–97 s) and was longer in patients with NCS without subsequent GCS than those with subsequent GCS [HR 2.276; CI 95% (1.565–3.311), *p* < 0.001] and in temporal lobe epilepsy compared to extratemporal [HR 1.753, CI 95% (1.065–2.885), *p* = 0.027]. ICA duration did not correlate with awake/sleep state at seizure onset (*p* > 0.050). Longer ICA duration was associated with lower SpO_2_ nadir during the NCS phase of seizures [HR 1.098, CI 95% (1.064–1.133), *p* < 0.001] and longer EEG recovery duration [HR 1.002, CI95% (1.001–1.003), *p* = 0.003]. ICA duration did not significantly correlate with hypoxemia duration, PGES duration, or SpO_2_ nadir in GCS.

Mean number of seizures per patient was 2.6 ± 1.7 (2; 1–8). In the total sample, 92/218 (42.2%) patients either had only one seizure, or only one analyzable seizure for ICA. The remaining 126/218 (57.8%) patients had recurrent seizures and comment on ICA could be made. ICA occurred in 60/126 (47.6%) patients, and recurred in 45/60 (75%) of the patients. No clinical characteristics (age, age at epilepsy onset, epilepsy duration, sex, and epileptogenic zone) were related to ICA recurrence (*p* > 0.05).

#### Clinical Case 1-Prolonged ICA and near-SUDEP

A 36 year-old right handed man with intractable right temporal lobe epilepsy of unknown etiology since the age of nine was enrolled into the study. His seizure semiology consisted of psychic aura followed by auditory aura with impaired awareness, and rare secondarily generalization. The last generalized convulsion had occurred 4 years before the admission. He had co-morbid depression. Previous antiepileptic drugs (AEDs) were carbamazepine, phenytoin, valproic acid and zonisamide. At admission for presurgical evaluation he was on oxcarbazepine 1,800 mg/day. Physical and neurological examinations were normal. Brain MRI was normal and the interictal PET scan showed bilateral mesial temporal hypometabolism, more pronounced on the right. Interictal recordings showed right temporal sharp waves (maximum at T8>F8). Retrospective review of older (non-study) VEEG records revealed a near-SUDEP incident (not included in the above analysis). The patient had 4 seizures during that admission. The first one, was a brief (<10 s duration auditory aura). The second and third seizures, were brief auras with rapid secondarily generalization, one arising from wakefulness and the other one arising from sleep. No comment about presence of ICA could be made on those seizures due to lack of plethysmography and rapid secondary generalization. No PCCA was noted in any of the GCS and regular breathing resumed immediately after clinical seizure end. These GCS occurred within 12 h of the fourth and last seizure. This was an apneic seizure with impaired awareness, and respiratory arrest lasting for 285 s, as evidenced by video analysis and oxygen desaturation. After a period of several shallow breaths, breathing finally resumed normally 311 s after seizure onset. Ictal EEG showed rhythmic alpha activity arising over the right antero-mesial temporal lobe with bilateral spread. No alteration in heart rhythm was noted apart from tachycardia. The patient was repositioned, oxygen administered, and ventilated with a face mask. He later underwent invasive evaluation, had further seizures without apnea, and a right temporal lobectomy in 2016 which resulted in seizure freedom (Engel Class 1, [>2 years]) Video 1.

### Post-convulsive Central Apnea (PCCA) Incidence, Duration, and Recurrence

Presence of PCCA could not be confidently ascertained in 9/237 (3.8%) GCS in 3/137 (2.2%) patients due to movement artifact. PCCA was present in 41/228 (18%) of GCS in 30/134 (22.4%) patients.

**Figure 3:**
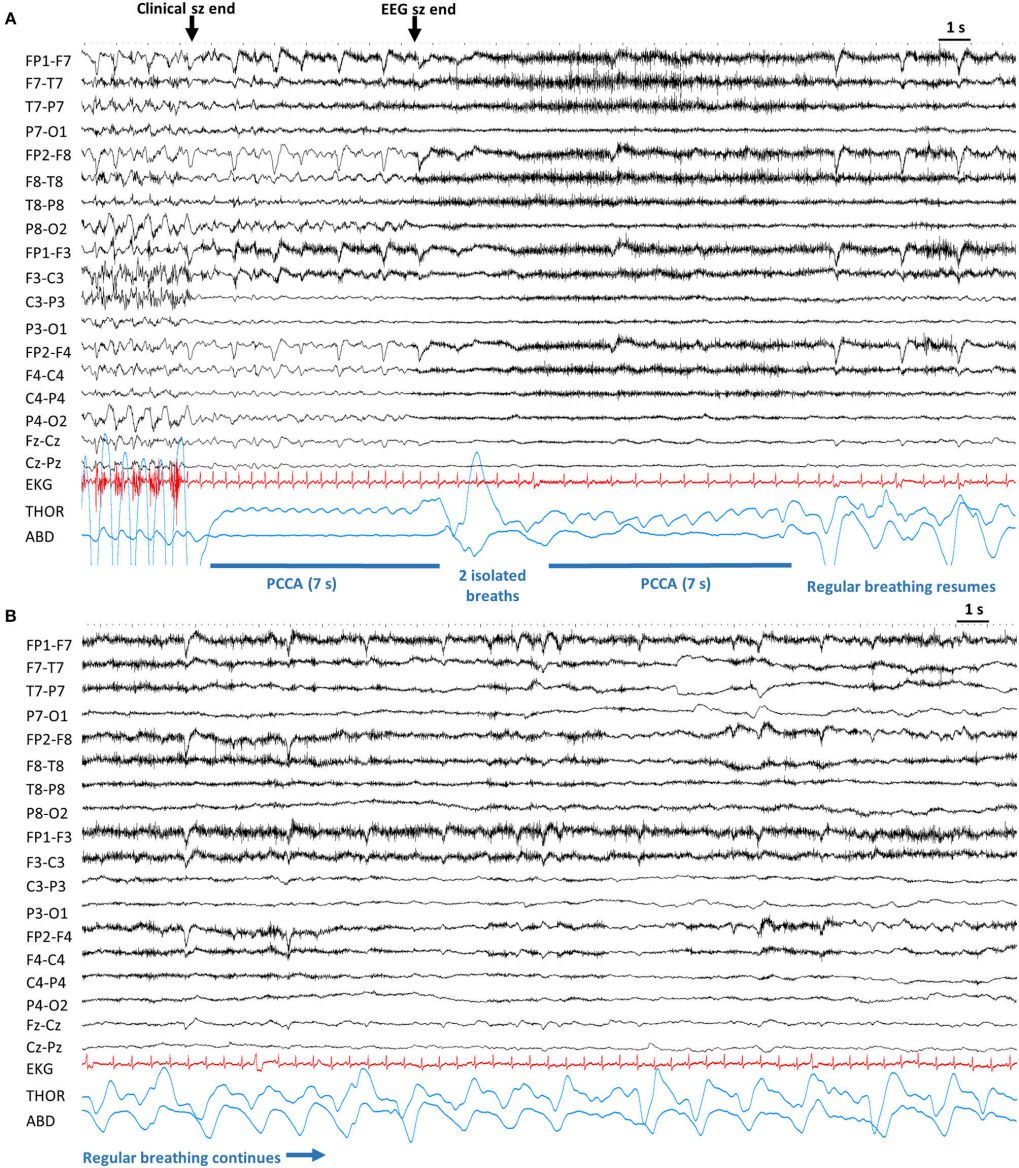
Example of post-convulsive central apnea (PCCA). Sensitivity 20 μV, High Frequency Filter: 70 Hz, Time constant: 0.1 s. **(A)**. After the end of convulsive phase, the patient is apneic for 7 s. After the electrographic end, there are 2 noticeable breaths which are followed by another brief apnea. Lastly, regular breathing resumes. **(B)** Continuation from **(A)**, regular breathing continues. ABD, abdominal; Clinical sz end, end of clinical seizure. EEG sz end, electrographic seizure end; EKG, electrocardiogram; PCCA, post-convulsive central apnea; s, seconds; THOR, thoracic.

In 24/41 (58.65%) seizures (in 19 patients), PCCA was observed without EEG seizure. In 14/41 (34.1%) seizures (in 12 patients), PCCA occurred with ongoing EEG seizure activity. In 3 seizures (in 3 patients) PCCA recurred in the same seizure, occurring initially with EEG seizure discharges and then after 1–2 breaths, recurring without accompanying seizure discharge(Figures 3A,B). In 13 seizures (in 11 patients), PCCA immediately followed clinical seizure end. In 25 seizures (in 20 patients), “delayed” PCCA occurred several breaths after clinical seizure end.

PCCA was more frequent in women than in men (*p* = 0.004) and occurred more often in generalized than focal epilepsies (*p* = 0.016). In focal epilepsy, it was more frequently seen in extratemporal than temporal (*p* = 0.020) patients, but there was no relationship with lateralization (*p* = 0.148). PCCA was unrelated to age at study or epilepsy onset, epilepsy duration and awake or sleep states (*p* > 0.050). Whereas, ICA presence was not related to PCCA occurrence, PCCA was significantly associated with longer ICA duration (*p* = 0.001).

Presence of PCCA was not related to PGES duration, and was not associated with EEG recovery duration and total hypoxemia duration (*p* > 0.050). However, PCCA was associated with longer SpO_2_ recovery times to mild hypoxemia (>90%). [RR 1.01, CI95% (1.003–1.017), *p* = 0.003] (Table 3).

**Table 3:** PCCA incidence and seizure characteristics.

			**Univariate analysis**	**Multivariate analysis**	
	**PCCA –**	**PCCA +**	***p***	**RR *(95% CI)***	***p***
Sex			0.004	11.29 (4.5–28.34)	<0.001
Male	103	10			
Female	84	31			
Age at study (yo)	37.7 ± 13.7	34.6 ± 14	0.267	–	–
Age at epilepsy onset	20.2 ± 17.2	19.5 ± 10.7	0.774	–	–
Epilepsy duration (y)	17.4 ± 12.1	14.9 ± 11.9	0.323	–	–
Epilepsy type			0.016	–	–
Generalized	25	11			
Focal	160	27			
Epileptogenic zone			0.020	4.48 (1.02–19.59)	0.046
Extratemporal	64	18			
Temporal	96	9			
Lateralization			0.148	–	–
Left	63	14			
Right	52	4			
State			0.738	–	–
Awake	97	20			
Asleep	90	20			
ICA duration	11.96 ± 5.75	18.6 ± 11.5	0.001	1.14 (1.05–1.25)	0.001
PGES^a^			0.091	0.27 (0.16–0.47)	<0.001
No	60	8			
Yes	126	33			
PGES duration^a^(s)	38.7 ± 18.9	39.5 ± 27.8	0.804	–	–
EEG recovery duration^a^ (s)	86.4 ± 80	83.5 ± 184.4	0.876	–	–
Recovery time to mild hypoxemia^a^ (s)	36.2 ± 31.3	58.3 ± 42	0.003	–	–
Total hypoxemia duration^a^ (s)	144.86 ± 70.3	139.1 ± 40.9	0.301	–	–
SpO_2_ nadir^a^	60.76 ± 13.8	58.3 ± 16	0.555	–	–

*GCS, generalized convulsive seizures; ICA, ictal central apnea; NCS, non-convulsive seizures; PCCA, post-convulsive central apnea; PGES, post-ictal generalized electroencephalographic suppression; SpO_2_, peripheral capillary oxygen saturation; y, years; y.o, years old*.

a *Analyzed only in GCS*.

After multivariate regression analysis, female sex [RR 11.297, CI 95% (4.50–28.34), *p* < 0.001] and ICA duration [RR 1.149 CI 95% (1.053–1.254), *p* = 0.001] were related to PCCA, whereas absence of PGES was related to absence of PCCA [RR = 0.274, CI 95% (0.159–0.471), *p* < 0.001].

Mean PCCA duration was 8.9 s ± 4.9 (5–32). PCCA duration was longer in males [HR 1.844, CI 95% (1.06–3.19), *p* = 0.003]. Epilepsy type, awake/sleep state did not influence PCCA occurrence. PCCA duration did not correlate with age, epilepsy duration, PGES duration, EEG recovery, hypoxemia duration, or time to recovery to mild hypoxemia (*p* > 0.050).

Mean number of GCS per patient was 1.7 ± 1 (1; 1–5). In patients with GCS, 77/137 (56.2%) had only one GCS and the remaining 60/137 (47.8%) had two or more GCS. In the group of patients with repeated GCS, comment on PCCA could be made on 57/60 (95%) patients. PCCA was seen in 17/57 (29.8%). In 9/17 (52.9%) patients, PCCA was recurrent.

#### Clinical Case 2-Prolonged PCCA and near-SUDEP

A 15 year-old right handed girl with epilepsy since age 5 years was admitted for evaluation. She was not an enrolled study patient. Seizures occurred once or twice a month and lasted up to two with whole body sensory aura (tingling) followed by oral automatisms with impaired awareness. This was rarely followed by secondary generalized convulsions lasting 1–2 min. On several occasions, paramedics were summoned as an emergency because of cyanosis and unresponsiveness after generalized convulsions. On admission she was on lamotrigine 200 mg/day and levetiracetam 3,000 mg/day, having previously failed multiple other AEDS. She had no epilepsy risk factors and no family history of epilepsy. Her physical and neurological examinations were normal. Epilepsy protocol MRI brain scans were normal on two occasions. Inter-ictal brain FDG-PET showed focal hypometabolism in the anterior left temporal lobe tip. Non–invasive VEEG monitoring showed left temporal sharp waves, maximum at F7/T7/FT9. Four habitual clinical seizures were recorded without secondarily generalization. EEG onsets were left hemispheric but not further localizable.

She underwent invasive EEG monitoring for better localization of the epileptogenic zone. A left subdural grid (8 × 6) was implanted along with strips covering the left orbitofrontal, superior temporal, inferior temporal regions, as well as left anterior-anterior, anterior-middle and anterior-posterior temporal, left middle temporal, left middle-middle, and middle-posterior. A left anterior temporal seizure was recorded, with typical automatisms and impaired awareness, right face clonic movements, and a secondary generalized tonic clonic seizure. After clinical seizure end, the patient was immediately apneic (as evidenced by video analysis, cyanosis, and severe O_2_ desaturation) for 126 s, followed by an isolated breath. A second period of apnea/hypopnea was then seen until regular breathing pattern resumed a total of 187 s after clinical seizure end. EEG seizure discharges were seen up to 25 s after clinical seizure end. Thirty nine seconds after clinical seizure end, there was concurrent progressive bradycardia followed by 10 s of asystole. Cardiac rhythm resumed, with a pattern of bradycardia and normal sinus rhythm, for 75 s, after which EKG signal was lost, but pulse artifact was evident on EEG. EEG suppression duration (all invasive electrodes), was ~254 s. During the episode, there was repeated tactile nursing intervention. Further, her head was re-positioned and O2 administered. No active resuscitation measures were performed. Due to continuing seizures, the patient underwent responsive neurostimulation (RNS® System) and was temporarily seizure free for 3 years, until recent recurrence of focal seizures at last follow up Video 2.

## Discussion

In this study we summarize incidence and risk factors for both ICA and PCCA. Additionally, we describe two near-SUDEP instances of prolonged ICA and PCCA, respectively.

ICA incidence in our study (43.2%), on a larger number of patients, was similar to those previously reported (10, 13). Consistent with our previous reports, ICA was not observed in patients with generalized epilepsy, and was more frequent in patients with temporal rather than extratemporal epilepsy (13, 14). ICA preceded other clinical signs in the vast majority of seizures and in almost half of them, also preceded EEG seizure onset. The observation of ICA being an exclusive feature for focal epilepsies, and especially in those from the temporal lobe, is consistent with previous human stimulation studies pointing out the amygdala, hippocampus, and mesial temporal pole, regardless of lateralization, as the symptomatogenic zone for ICA (11, 13, 22). The absence of ICA in the GCS of generalized epilepsy is in large part due to immediate or rapid generalization where breathing compromise may be partly or wholly due to generalized muscle tonicity that includes respiratory musculature, rather than solely due to unequivocal central apnea. However, we cannot be sure that these patients truly do not have ICA.

Unlike previous publications (13), where no differences between awake/sleep states at seizure onset were found, in this more robustly powered study, ICA occurred more frequently in seizures arising from sleep. One possible explanation is that ICA is easier to detect in the sleep state, where acquisition artifact is less prevalent. However, awake recordings were not disproportionately excluded because of artifact and such disparities can be explained by physiological differences in breathing control during sleep and wakefulness (23). Breathing is under automatic control through multiple pontomedullary nuclei, the pre-Bötzinger complex (pre-BötC) comprising the main rhythm generator (24). Cortical and subcortical structures, such as thalamus, hypothalamus, amygdalo-hippocampal complex, cerebellum, and mesencephalic nuclei relay to pontomedullary respiratory centers and along with peripheral sensory feedback, modulate breathing output (25). There is increased evidence that serotoninergic neurons lying in the midline raphe nuclei play an active role in both arousal and chemoreception (26, 27). These neurons tonically excitate multiple components of the brainstem respiratory network, with interconnections with the pre-BötC, and also act as central chemosensors, detecting changes in tissue CO_2_/H^+^ modulating the aforementioned tonic excitatory drive to adjust ventilation accordingly (28). Mice with genetically deleted medullary serotoninergic neurons lack any arousal response to inhalation of CO_2_ but have normal arousal responses to other stimuli such as hypoxia, sound and air puffs (26). Moreover, the activity of medullary raphe serotoninergic neurons is highest during wakefulness and absent during REM sleep (28). Most SUDEP cases occur at night, and in the MORTEMUS study this was true in the majority of monitored cases (5) animal studies reveal brainstem serotoninergic dysfunction during and after seizures, with decreased firing of the medullary raphe neurons during the ictal and post-ictal periods (29). Therefore, in the setting of a potentially dysfunctional serotoninergic network in epileptic patients, sleep would constitute a vulnerable period for breathing disturbances (30).

The near-SUDEP case with prolonged ICA, in whom breathing resumed after seizure end raises a number of interesting issues. First, it seems possible that unobserved and in the absence of active intervention, the outcome could have been fatal. This supports the contention that prolonged ICA is dangerous and potentially lethal. Second, the near-fatal seizure episode may be more akin to focal status epilepticus, and supports the view that the latter cannot be excluded in at-home, unobserved deaths that are usually labeled SUDEP (31). Revisiting SUDEP definition (which mandates exclusion of status epilepticus) may be necessary. The episode mimics a sheep model of status epilepticus and apneic death (32). Third, seizure termination and resumption of breathing with seizure end, suggest that ICA is driven by seizure discharge (likely in the mesial temporal structures), rather than other mechanisms (13). Finally, why the majority of ICA instance are self-terminating and some become prolonged, is unresolved, but may reflect the consequences of damage caused by early onset, long-standing epilepsy, and frequent GCS to key breathing control sites (amygdala, hippocampus, dorsal thalamus, anterior cingulate, ventrolateral medulla etc.) (33–35), rendering greater susceptibility to exaggerated apneic responses.

PCCA incidence (22.4%) was almost half of ICA incidence, more frequently observed in female subjects (although more likely to be longer in male patients), and commoner in those with longer ICA duration. In contrast to ICA, PCCA was observed in both patients with focal, and generalized epilepsy. These differences suggest differing pathophysiologies (14). Whereas, ICA appears to be a semiological phenomenon most often resulting from seizure activity in the amygdalo-hippocampal complex, PCCA most likely results from seizure spread to the brainstem during GCS, regardless of epileptogenic zone (36). Breathing cessation may be derived either from active depolarization and activation of crucial breathing centers that generate apneic responses, such as the periaqueductal gray (37, 38), or disruption of the normal functioning of rhythm-generating neurons and its intricate network, leading to breathing cessation (39). This is consistent with animal models of SUDEP showing post-ictal depolarization in dorsal medulla (40), in which apnea and PGES precede cardiac arrest, and resemble the clinical phenotype of monitored SUDEP patients in the MORTEMUS study (5). Human neuroimaging and neuropathological studies have shown damage in key brainstem structures that modulate breathing, such as the medullary raphe and ventrolateral medulla, in SUDEP and high SUDEP-risk patients (33, 41, 42).

In our study, ICA duration conferred higher risk for PCCA and was related to lower SpO_2_ nadirs in the pre-convulsive phase. This may indicate greater hypoxemia induced brainstem compromise, leading to PCCA. Seizure induced focal brainstem hypoxia, due to vasospasm, has been described in animal models and posited as a potential mechanism for SUDEP (43–45). Moreover, we found that the presence of PCCA was related to longer hypoxemia recovery times (SpO_2_ > 90%). Although causality could not be established, longer hypoxemia recovery times may be a consequence of PCCA. However, PCCA duration itself, was not related to hypoxemia severity or duration. PCCA durations were typically short, and hypoxemia severity may be more related to GCS severity, although PCCA impact on hypoxemia may become independently important in instances of prolonged PCCA.

Although PCCA occurred preferentially in women, its duration was longer in men, consistent with the SUDEP phenotype (2). Sex differences in breathing function and the protective role of estrogens in respiratory diseases may explain these findings (46–48), since differences in epilepsy phenotype do not explain these differences. As with ICA, duration rather than presence, may primarily influence SUDEP risk in PCCA.

PGES is a frequent finding in GCS, particularly in those arising from sleep and is related to the symmetric tonic phase, postictal immobility, lack of early oxygen administration, duration of oxygen desaturation and lower SpO_2_ nadir values (49–51). PGES has been postulated as a SUDEP biomarker, especially if prolonged (>50 s) (18). Its relationship with ICA or PCCA has not been established, except indirectly through O_2_ desaturation findings (50). In our study, PCCA was proportionally seen more frequently in seizures with PGES than in seizures without. The pathogenesis of PGES is not well-determined. Cortical neuronal exhaustion or a disruption of ascending inputs after a GCS, or a combination of both, are viable hypotheses. Disruption of ascending pathways such as from the reticular activating system may conceivably prolong the comatose post-ictal state, as well as modulate cortical neuronal activity, and thus, impair the protective behavioral effect of arousal to overcome PCCA (52).

ICA recurred in 75% of the cases, whereas PCCA recurred in 52.9%. This may further reinforce that ICA is a semiological, and therefore recurrent, phenomenon. However, PCCA seems only slightly less frequent, and may also be semiological rather than probabilistic, although our two case reports suggest that prolonged instances of either, are what potentially determine mortality risk. PCCA instances combined with bradycardia/asystole may be particularly dangerous. Our second clinical case of prolonged PCCA accompanied by asystole, resembles the clinical phenotype described in the MORTEMUS study and in a recent analysis of this cohort in a smaller number of patients (14). Invasive monitoring did not show ongoing seizure activity that was concurrent with apnea, reinforcing once again, the different pathophysiologies of ICA, and PCCA, with higher likelihood of involvement of subcortical structures, such as the brainstem, in PCCA (53, 54).

Our study has several limitations. First, it is an observational study in a select group of patients (i.e., primarily treatment resistant epilepsy) and does not necessarily reflect seizure phenomenology or SUDEP risk in a treatment responsive population. Detection of ICA was heavily dependent on extent of acquisition artifact, and hence we may have underestimated incidence. Alternatively, postictal immobility after GCS allowed PCCA identification in the majority of cases. Breathing analysis through polygraphic study was limited to thoraco-abdominal movement and pulse oximetry. Thus, additional information on the presence of mixed central/obstructive apneas, is unavailable. Our apnea definition differs from previous literature, which is based on the 10 s sleep-study criterion, and therefore, ICA and PCCA incidence may be overestimated in our study. However, based on our brain stimulation experiments on breathing modulation, where brief stimulation periods result in immediate and brief (<10 s) apneas, we believe our definition is both accurate and sensitive (12, 22). Our conclusions are based on a relatively small group of number seizures in the primary generalized epilepsy group compared to patients with focal epilepsy. Lastly, our study was based on surface EEG and persistence of intracranial seizure in deep, apnea causing structures (12) cannot be completely excluded in patients with PCCA. However, Case 2 above, along with a previous case reported in literature, suggest that apnea in epileptic patients can occur in the absence of electrographic seizure (53).

## Conclusions

Peri-ictal central apnea takes two main forms, as ICA or PCCA. ICA incidence is almost twice PCCA incidence and is only seen in focal epilepsies, suggesting different pathophysiologies. Both ICA and PCCA may be recurrent, but prolonged instances leading to SUDEP and near-SUDEP may be probabilistic instances. Prolonged ICA is related to presence of PCCA, possibly due to greater effect of ICA-induced hypoxemia on brainstem function. Absence of PCCA is associated with absence of PGES, suggesting that PCCA presence directly correlates with GCS severity. Alternatively, brainstem structures responsible for arousal and breathing may obviate PGES occurrence. Apnea preceding both EEG as well as clinical seizure onset in a substantial number of patients suggests that plethysmographic respiratory monitoring in regular clinical practice may have seizure detection value. Moreover, such monitoring may facilitate detection of prolonged ICA and PCCA, thus allowing SUDEP risk quantification although further evidence is required to confirm this. Both prolonged ICA and PCCA may contribute to SUDEP. Further prospective cohort studies are needed to validate these hypotheses.

## Data Availability

The datasets analyzed in this study are available from the corresponding author on request.

## Author Contributions

LV had a major role in the acquisition and analysis of data, interpreted the data, drafted the manuscript for intellectual content. NL designed and conceptualized study, interpreted the data, revised the manuscript for intellectual content. JH analysed the data, performed statistical analysis, images and video editing. MR acted as a recruiter, revised the manuscript for intellectual content. KL analysed the data. RS, DF, MN, BG, SS, JO, RH, BD, LB, OD, and GR revised the manuscript for intellectual content. KS interpreted the data, revised the manuscript for intellectual content. LA, CS, BZ, NH, NS, XZ, VR-M, and AT performed data acquisition. AZ led and coordinated communication among sites; revised the manuscript for intellectual content. CT analysed the data and performed statistical analysis. SL designed and conceptualized study, performed analysis and interpretation of data, revised the manuscript for intellectual content.

### Conflict of Interest Statement

The authors declare that the research was conducted in the absence of any commercial or financial relationships that could be construed as a potential conflict of interest.
